# Clinical application value of metagenome next-generation sequencing in pulmonary diffuse exudative lesions: a retrospective study

**DOI:** 10.3389/fcimb.2024.1367885

**Published:** 2024-05-08

**Authors:** Yisong Wu, Jian Wu, Nengluan Xu, Ming Lin, Wenxiang Yue, Yusheng Chen, Qiongyao Zhang, Hongru Li

**Affiliations:** ^1^ Fujian Provincial Hospital, Shengli Clinical College of Fujian Medical University, Fuzhou, Fujian, China; ^2^ Department of Infectious Diseases, Fujian Provincial Hospital, Shengli Clinical College of Fujian Medical University, Fuzhou, Fujian, China; ^3^ Fujian Provincial Key Laboratory of Medical Big Data Engineering, Fujian Provincial Hospital, Fuzhou, Fujian, China

**Keywords:** metagenomic next-generation sequencing, pulmonary diffuse exudative lesions, respiratory pathogens, prognostic value, diagnostic value

## Abstract

**Objective:**

This study aims to investigate the clinical application value of Metagenome Next-Generation Sequencing (mNGS) for pulmonary diffuse exudative lesions.

**Methods:**

From January 1, 2014, to November 31, 2021, 136 cases with chest radiologic presentations of pulmonary diffuse exudative lesions admitted to Fujian Provincial Hospital were included in the study; of those, 77 patients underwent mNGS pathogen detection. Based on the pathogen detection outcomes and clinical diagnoses, patients were categorized into an infection group (IG) and a non-infection group (NIG). A comparison was made between the diagnostic efficacy of the mNGS technique and traditional culture methods. Meanwhile, 59 patients clinically identified as having infectious pulmonary diffuse exudative lesions but who did not receive mNGS testing were designated as the non-NGS infection group (non-IG). A retrospective cohort study was conducted on patients in both the IG and non-IG, with a 30-day all-cause mortality endpoint used for follow-up.

**Outcomes:**

When compared to conventional culture methods, mNGS demonstrated an approximate 35% increase in sensitivity (80.0% vs 45.5%, P<0.001), without significant disparity in specificity (77.3% vs 95.5%, P=0.185). Under antibiotic exposure, the positivity rate detected by mNGS was notably higher than that by traditional culture methods, indicating that mNGS is less affected by exposure to antibiotics (P<0.05). Within 30 days, the all-cause mortality rate for patients in the IG versus the non-IG was 14.55% and 37.29%, respectively (P<0.05). Following a COX regression analysis to adjust for confounding factors, the analysis revealed that a CURB-65 score ≥3 points (HR=3.348, P=0.001) and existing cardiovascular disease (HR=2.473, P=0.026) were independent risk factors for these patients. Conversely, mNGS testing (HR=0.368, P=0.017) proved to be an independent protective factor.

**Conclusion:**

mNGS technology makes it easier to pinpoint the cause of pulmonary diffuse infectious exudative lesions without much interference from antibiotics, helping doctors spot and diagnose these issues early on, thereby playing a key role in helping them decide the best treatment approach for patients. Such conclusions may have a bias, as the performance of traditional methods might be underestimated due to the absence of complete results from other conventional diagnostic techniques like serological testing and PCR.

## 1 Introduction

Pulmonary diffuse exudative lesions refer to a condition where a patient’s chest radiography reveals unilateral or bilateral lungs manifesting diffuse exudative changes, often affecting multiple lobes. Clinicians commonly characterize these alterations as “white lung” patterns. These disorders are distinguished by abrupt onset, rapid progression, intricate etiologies, and considerable prognostic variability among patients. However, given that the term “acute pulmonary diffuse exudative lesion” has only recently emerged in medical parlance, the current discourse and research on this category of illness remain scant (Lozano et al., 2012; Oliveira et al., 2018). In recent years, the expansive application of corticosteroids, antibiotics, immunosuppressants, chemotherapeutic agents, as well as radiotherapy, along with the continual emergence of novel pathogens and respiratory infections, has led to a steady increase in the number of patients afflicted by these conditions, while simultaneously compounding the complexity of etiologies and clinical presentations. In the treatment of non-infectious pulmonary diffuse exudative lesions, it is imperative to modulate the regimen of corticosteroids and immunosuppressants. Empirical administration of antibiotics in large quantities without identifying the infectious agent does not aid in ameliorating the condition, and may, in fact, exacerbate antibiotic misuse and delay appropriate treatment. On the other hand, given the rapid progression of infectious pulmonary diffuse exudative lesions, the absence of timely and precise anti-infection treatment could precipitate the aggravation of the infection in a brief span, potentially leading to respiratory failure, escalated systemic infection, multiple organ dysfunction syndrome, or even death. Thus, it is of paramount significance to accurately discern between infectious and non-infectious diseases at an early stage of illness, and to promptly identify the causative agent of the infectious disease. This enables the selection of an appropriate and targeted treatment plan, which is crucial for enhancing the patient’s prognosis.

Presently, the etiological diagnosis of patients with pulmonary diffuse exudative lesions still relies on conventional pathogen detection protocols. These primarily encompass methods such as smear microscopy, isolation and culture techniques, immune assays related to antigen-antibody interactions, and molecular biological techniques targeting specific nucleic acid sequences of pathogens, including Polymerase Chain Reaction (PCR) and its derivative technologies. Conventional culturing methods are beset by considerable limitations, such as protracted detection cycles, a narrow spectrum of detectable pathogens, and low positivity rates (Zandstra and van der Voort, 2004; Zhang et al., 2020) Furthermore, the fraction of pathogenic microorganisms that are amenable to culture constitutes a mere 1% (Daniel, 2005), leaving a substantial proportion of pathogens which are recalcitrant to cultivation. Furthermore, the administration of antibiotics affects the positive rate of traditional cultures (Afshar et al., 2009; Miao et al., 2018), and especially in patients with severe conditions, prior exposure to antibiotics before sample collection could potentially influence the final diagnostic outcome. Alternative detection methods also come with their inherent limitations. The PCR technique necessitates pre-screening of pathogens, along with the design of specific primers or probes, thus demanding sophisticated expertise, and it permits the identification of only a finite array of pathogens; serological antibody testing is retrospective in nature, lacking the timeliness required for immediate diagnosis. Consequently, there is a pressing need for diagnostic methodologies that are faster, more comprehensive, and more precise in order to facilitate the early diagnosis of etiological agents, enabling prompt and targeted treatment to ameliorate patient outcomes.

Metagenomic Next-Generation Sequencing (mNGS) is a pathogen diagnostic technology that has developed in recent years (Wilson et al., 2014). It allows for the direct acquisition of pathogen nucleic acid sequence information from the detection sample. By comparing and analyzing with all the pathogenic microorganism sequences contained in the database, mNGS can unbiasedly detect all pathogenic microorganisms in the detection sample in a short period of time with the advantage of having a wide range of detectable microorganisms. In addition, it can also detect potential and rare microorganisms that are difficult to detect using traditional detection methods, providing more powerful evidence for clinical diagnosis and treatment. Furthermore, mNGS exhibits less susceptibility to antibiotic exposure compared to traditional culture methods (Li et al., 2018). Additionally, mNGS offers a shorter detection time, with an average of 48 hours, significantly less than traditional culture methods (Simner et al., 2017; Brown et al., 2018). Due to its unbiased nature, high throughput capabilities, broad detection range, and rapid efficiency, mNGS has been widely applied in various domains of clinical medicine (Diseases, 2020). These include not only respiratory (Walker et al., 2015; Sun et al., 2021; Yang et al., 2021), digestive (Lewandowska et al., 2015), urinary tract (McDonald et al., 2017), and central nervous system (Wilson et al., 2014; Guan et al., 2015; Wang et al., 2019b) related infectious diseases, but also research in drug-resistant gene detection and cancer gene detection (Guo et al., 2021). However, mNGS also suffers from being costly and detecting a plethora of pathogenic species, making it difficult to identify the causative agent, thereby limiting its widespread application. Currently, there is limited research on the application of mNGS in the diagnosis and treatment of diffuse infiltrative lung lesions, and the impact of mNGS technology on the prognosis of such patients remains unclear. Therefore, it is necessary to analyze and explore the value of mNGS in the application of diffuse infiltrative lung lesions. In this study, a retrospective analysis was conducted to collect and analyze data from patients with diffuse infiltrative lung lesions admitted to Fujian Provincial Hospital from January 1, 2014 to November 31, 2021, aiming to ascertain the value of mNGS in the diagnosis of clinical infectious diseases.

## 2 Materials and methods

### 2.1 Subject

**Figure 1:**
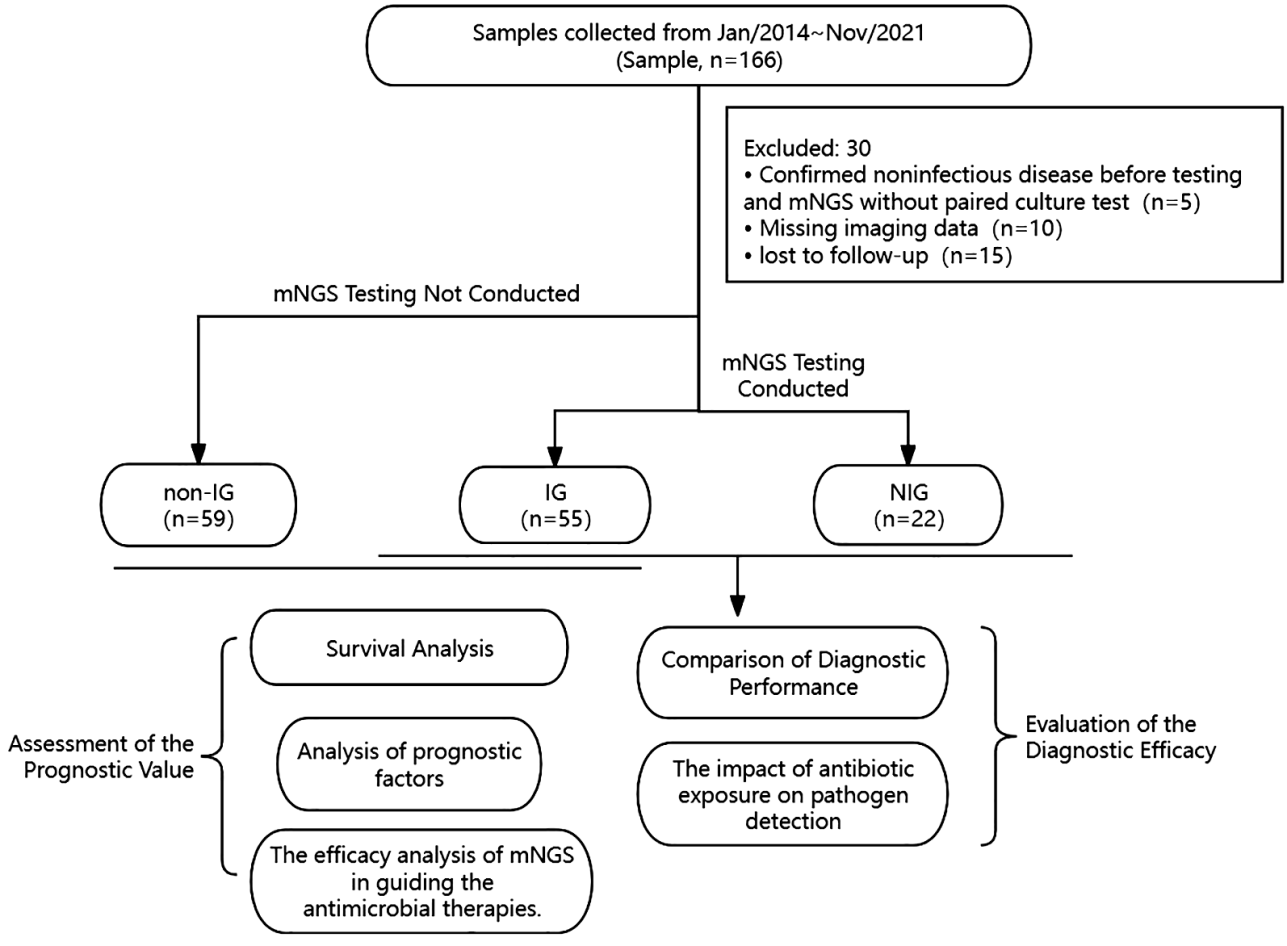
Flowchart of sample selection, classification, and comparison. From 166 samples, a total of 136 were selected for further analysis. Samples were divided into infection group (IG), non-infection group (NIG) and non-NGS infection group (non-IG). Based on the pathogen detection outcomes and clinical diagnoses, 77 patients were categorized into an infection group (IG) (n=55) and a non-infection group (NIG) (n=22). Besides, 59 patients clinically identified as having infectious pulmonary diffuse exudative lesions but who did not receive mNGS testing were designated as the non-NGS infection group (non-IG). Between IG and NIG, we conducted a comparison of the diagnostic capabilities of mNGS with conventional culture methods for pulmonary diffuse exudative lesions. Meanwhile, between IG and non-IG, we assessed the prognostic value of mNGS versus traditional culturing techniques for infectious pulmonary diffuse exudative lesions.

A total of 166 cases with pulmonary infiltrates, either unilateral or bilateral, were collected from January 1, 2014, to November 31, 2021, in Fujian Provincial Hospital, and they met the inclusion criteria for hospitalization. After excluding 30 cases based on the exclusion criteria, a final number of 136 cases were included in this study. Among the 77 patients with pulmonary diffuse exudative lesions who underwent metagenomic next-generation sequencing (mNGS) testing, they were categorized based on pathogen detection results and clinical diagnoses into an infection group (IG) (n=55) and a non-infection group (NIG) (n=22). All patients had results from conventional culture methods. The diagnostic efficacy of mNGS testing versus traditional culture was comparatively evaluated between the IG and NIG, analyzing the advantages of mNGS in diagnosing the etiology of pulmonary diffuse exudative lesions. Among the cohort meeting the inclusion criteria, 59 patients were clinically diagnosed with infectious pulmonary diffuse exudative lesions but did not undergo metagenomic next-generation sequencing (mNGS) testing. These patients were categorized as the non-NGS infection group (non-IG) (n=59). A 30-day follow-up was conducted to compare and analyze the disparities in survival rates at 30 days, the efficacy of therapeutic adjustments, and potential independent prognostic factors between the non-IG and the IG subjected to testing. (See **Figure 1** for details).

Inclusion criteria: 1. Age ≥ 18 years. 2. Chest imaging indicative of unilateral or bilateral pulmonary diffuse infiltrative lesions. 3. Patient informed consent obtained and the informed consent document duly signed.

Exclusion criteria include: 1. Individuals under the age of 18; 2. Conditions such as pulmonary edema, pulmonary hemorrhage, diffuse alveolar damage, pulmonary alveolar proteinosis, hypersensitivity pneumonitis, eosinophilic pneumonia, bronchioloalveolar carcinoma, and infiltrative mucinous adenocarcinoma, which can present as diffuse abnormalities on imaging studies; 3. A substantial deficit in clinical data; 4. Patients lost to follow-up; 5. Cases where the final clinical diagnosis is non-infectious and molecular next-generation sequencing (mNGS) tests have not been conducted.

### 2.2 Data collecting

For the patients enrolled in our study, we have amassed a plethora of clinical data, including their history of antibiotic exposure in the seven days prior to admission, radiological examinations, conventional culture results (sputum, blood, bronchoalveolar lavage fluid, lung tissue, pleural effusion), results of metagenomic next-generation sequencing (mNGS), time required for both mNGS and traditional culture tests, CURB-65 scores, antimicrobial treatment regimens, clinical outcomes, and follow-up results. Subsequent to this gathering of information, we conducted pertinent statistical analyses.

Potential research variables that may be subject to ambiguity are clarified as follows:

1) History of antibiotic exposure within seven days prior to admission: Treatment with one or more antibiotics within the seven days preceding hospital admission, with a minimum duration of therapy extending at least 48 hours.
2) Duration of testing: Irrespective of whether metagenomic next-generation sequencing (mNGS) or conventional culture testing is employed, the calculation of the testing duration commences with the initiation of the sample collection and extends to the point at which the results are reported.
3) Criteria for Positive mNGS Outcomes: 1. Bacteria and viruses (excluding mycobacteria): Per Langelier’s study (Langelier et al., 2018), mNGS identifies a bacterial or viral organism at the species level when the sequence count supersedes the cumulative count of all other microbes by a tenfold margin; 2. Fungi: The detection of a fungal species is deemed significant by mNGS when its sequence count quintuples that of all other fungi; 3. Mycobacteria: i. Mycobacterium tuberculosis (MTB): A positive indication through mNGS requires the identification of at least one sequence attributable to the Mycobacterium tuberculosis complex at the genus level; ii. Nontuberculous mycobacteria (NTM): Due to potential environmental contamination, a positive result is acknowledged when sequences of NTM, at either the genus or species level, rank among the top ten bacterial counts.
4) The CURB-65 score: 1. Confusion of new onset, 2. Blood urea nitrogen levels >7 mmol/L (19 mg/dL), 3. A respiratory rate equal to or exceeding 30 breaths per minute, 4. Systolic blood pressure ≤90 mmHg or diastolic blood pressure ≤60 mmHg, 5. An age of 65 years or older. Each criterion met corresponds to one accumulated point, culminating in a total score.
5) The efficacy or ineffectuality of adjustments: The final verdict was ascertained following a comprehensive assessment, conducted by two seasoned pulmonologists at or above the level of associate director. They evaluated both the efficacy of the adjusted treatment and the resultant progression of the patient’s condition.

### 2.3 Operation and analysis steps of metagenomic next-generation sequencing

1) Specimen Processing and Nucleic Acid Extraction:
a) Blood samples: Acquire 3 ml of blood, and within 8 hours, subject the collection tube to centrifugation at 4°C and 2910 rcf for 10 minutes. Harvest 300 µl of plasma, and utilize the TIANamp Micro DNA Kit (DP316, TIANGEN BIOTECH, Beijing, China) to extract DNA according to the manufacturer’s protocol provided with the kit. The extracted DNA will be utilized for DNA library construction as referenced in (Long et al., 2016).
b) Bodily fluid samples: Following standard collection protocols, gather 1.5-3 ml of patient’s sputum, bronchoalveolar lavage fluid (procedures for other specimens such as tissues, cerebrospinal fluid, etc., are similar to the aforementioned), take 600 µl, and mix with 250 µl of 0.5 mm glass beads for mechanical lysis. Then add 7.2 µl of Lyticase (RT410-TA, TIANGEN BIOTECH, Beijing, China) to proceed with enzymatic lysis. After thorough mixing and agitation, retrieve 300 µl of the sample and proceed with DNA extraction using the TIANamp Micro DNA Kit (DP316, TIANGEN BIOTECH, Beijing, China) following the steps outlined in the reagent kit.
2) Library Construction and Sequencing: Enzymatic fragmentation, end repairing, adaptor ligation, and PCR amplifications are carried out on the extracted nucleic acids to construct the library. The library fragment size is quality-controlled at around 300bp using an Agilent 2100 Bioanalyzer, and the DNA library concentration is controlled using the Qubit dsDNA HS Assay Kit (Thermo Fisher Scientific Inc.). Post-assessment, the libraries are pooled in equimolar amounts and the pooled libraries are then circularized to form single-stranded circular structures. These are followed by rolling circle amplification (RCA) to generate DNA nanoballs (DNBs). The DNBs thus prepared are loaded onto sequencing chips and sequenced using the BGISEQ-50/MGISEQ-2000 platforms (Jeon et al., 2014).
3) Data Analysis: Post-sequencing, low-quality sequences are removed to obtain high-quality data. The high-quality data are aligned to the human reference genome by BWA (BWA: http://bio-bwa.sourceforge.net/) to discard any sequences that match, excluding them from the dataset (Li and Durbin, 2009). The remaining data, after low-complexity reads have been excluded, are matched against the PMDB pathogen database (which includes 6,350 bacteria, 1,064 fungi, 4,945 viruses, and 234 parasites), yielding the number of sequences that align with each type of pathogen. The quantity of sequence matches, along with other clinical test results, are then used to infer the probable pathogens.

### 2.4 Statistical methods

Statistical analyses were performed using the SPSS 26.0 software suite. Preliminary assessments included testing for normality and homogeneity of variance in continuous data. Metrics adhering to a normal distribution are expressed as the mean ± standard deviation, while those not conforming to normality are represented using the median and interquartile range. For comparisons of normally distributed quantitative data between groups, the t-test or a corrected t-test was employed. In contrast, the Mann-Whitney U test was utilized for data that deviated from a normal distribution. Categorical data were denoted using frequencies and percentages (%), and comparisons between groups were conducted using the Chi-square test or Fisher’s exact test, as appropriate. All statistical tests were two-tailed, with a p-value of less than 0.05 considered to indicate statistical significance.

## 3 Results

### 3.1 Baseline data

The study encompassed a total of 136 subjects, divided into three cohorts: the IG (n=55), the non-IG (n=59), and the NIG (n=22). Within the IG of 55 individuals, there were 41 males (74.55%) and 14 females (25.45%), with a median age of 65.00 years (interquartile range [IQR], 57.00–72.00 years). The non-IG comprised 59 subjects, which included 44 males (74.58%) and 15 females (25.42%), with a median age of 67.00 years (IQR, 56.00–75.00 years). The NIG, tallying 22, consisted of 12 males (54.55%) and 10 females (45.45%), with a median age of 60.50 years (IQR, 46.75–64.25 years).

Comparative analyses between the IG and non-IG revealed no significant statistical difference in variables such as gender, age, cardiovascular diseases, renal disorders, hepatic conditions, malignancies, diabetes, rheumatic immune diseases, and smoking habits (P > 0.05, as detailed in **Supplementary Table 1**). In the NIG, diagnoses for each patient are detailed in **Supplementary Table 2**.

### 3.2 The comparative efficacy of mNGS and traditional culture techniques in diagnosing pulmonary diffuse exudative lesions

#### 3.2.1 Comparison of diagnostic performance

**Figure 2:**
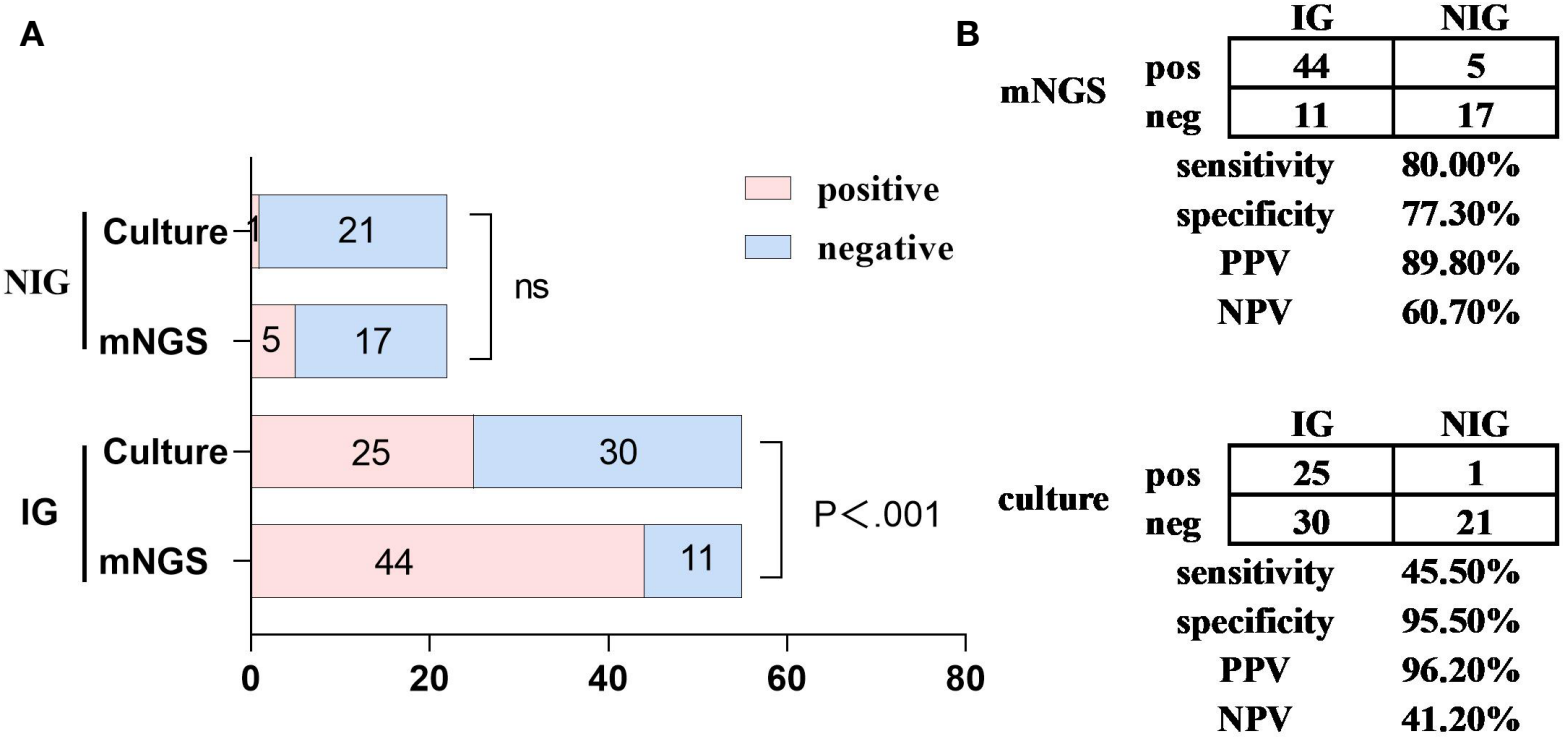
A comparative analysis of the capabilities of mNGS and traditional culture diagnostics for pulmonary diffuse infiltrative lesions. Metagenomic Next-Generation Sequencing (mNGS) versus culture methods for the detection of infectious diseases (IG, n=55) and non-infectious diseases (NIG, n=22). **(A)** the number of positive samples (x-axis) identified by paired mNGS and culture methods is charted according to IG and NIG cases. **(B)** a contingency table presents the diagnostic capabilities of mNGS and culture methods in differentiating between IG and NIG in a 2x2 format. Compared to culture methods, mNGS demonstrated an approximate 35% increase in sensitivity (80.0% vs 45.5%, P<0.001), whereas the specificity showed no significant statistical difference. IG, infectious disease; mNGS, metagenomic next-generation sequencing; NIG, noninfectious disease; NPV, negative predictive value; ns, no significant difference; pos, positive; neg, negative; PPV, positive predictive value.

The negative predictive value and positive predictive value of mNGS for diagnosing infectious pulmonary diffuse exudative lesions are 60.7% and 89.8%, respectively, while the negative likelihood ratio and positive likelihood ratio are 0.26 and 3.52, respectively. Compared to traditional culture methods, mNGS has demonstrated an approximate 35% increase in sensitivity (80.0% vs 45.5%, P < 0.001) with no significant difference in specificity (77.3% vs 95.5%, P = 0.185) (see **Figure 2**).

#### 3.2.2 The impact of antibiotic exposure on pathogen detection

Among a cohort of 77 patients consisting of both IG and NIG, 64 patients had received at least one course of antibiotic treatment within the 7 days preceding their admission, with the duration extending beyond 48 hours. The remaining individuals had no prior exposure to antibiotics. In patients without a history of antibiotic exposure, the positive detection rates between mNGS and conventional culture methods did not exhibit a significant disparity (30.77% vs. 0%, P=0.096). Conversely, among the patient subset with previous antibiotic exposure, mNGS demonstrated a significantly higher positivity rate compared to traditional cultures, a discrepancy backed by statistical significance (70.31% vs. 40.63%, P<0.001, as illustrated in **Supplementary Figure 1**).

### 3.3 The clinical value of mNGS compared to conventional culture methods for infectious pulmonary diffuse exudative lesions

#### 3.3.1 Clinical data supplementation for the IG group versus the non-IG group

The general conditions of the IG and the non-IG, as well as their comparative analysis, are presented above (**Table 1** and **Supplementary Table 1**). The clinical manifestations and auxiliary examination results of both groups are depicted in the following **Table 2**. A statistically significant difference between the two cohorts was observed solely in the levels of aspartate aminotransferase (AST), with a p-value of less than 0.05.

**Table 1:** Baseline characteristics of the included patients.

	IG (n=55)	non-IG (n=59)	NIG (n=22)
Age	65.00 (57.00, 72.00)	67.00 (56.00, 75.00)	60.50 (46.75, 64.25)
Sex			
Male	41/55 (74.55%)	44/59 (74.58%)	12/22 (54.55%)
Female	14/55 (25.45%)	15/59 (25.42%)	10/22 (45.45%)
Cardiovascular diseases	24/55 (43.64%)	33/59 (55.93%)	5/22 (22.73%)
Chronic kidney disease	10/55 (18.18%)	10/59 (16.95%)	0/22 (0.00%)
Chronic liver diseases	10/55 (18.18%)	5/59 (8.47%)	2/22 (9.09%)
Autoimmune rheumatic disease	9/55 (16.36%)	9/59 (15.25%)	9/22 (40.9%)
Tumor	8/55 (14.55%)	6/59 (10.17%)	2/22 (9.09%)
Diabetes	16/55 (29.09%)	14/59 (23.73%)	1/22 (4.55%)
Smoking	25/55 (45.45%)	29/59 (49.15%)	5/22 (22.7%)

**Table 2:** Comparative analysis of clinical data between the IG and non-IG.

	IG (n=55)	non-IG(n=59)	χ^2^/t/U	P-value
Fever	44/55(80.00%)	48/59(81.36%)	0.034	0.855
Cough	44/55(80.00%)	50/59(84.75%)	0.443	0.506
Expectoration	40/55(72.73%)	44/59(74.58%)	0.050	0.823
Dyspnea	35/55(63.64%)	42/59(71.19%)	0.740	0.390
PCT (ng/ml)	0.45 (0.25, 5.30)	1.82 (0.32, 7.16)	1391	0.243
CRP (mg/L)	145.00±13.56	152.50±12.74	0.404	0.687
WBC (10^9/L)	10.92±0.72	11.72±0.88	0.702	0.484
Hb (10^9/L)	115.40±3.31	115.50±3.38	0.034	0.973
BUN (mmol/L)	5.80 (3.80, 9.90)	8.30 (5.20, 10.60)	1280	0.052
ALT (IU/L)	29.00 (14.00, 48.00)	25.00 (19.00, 47.50)	1483	0.623
AST (IU/L)	33.00 (22.00, 47.00)	48.00 (27.50, 90.00)	1140	0.013
T.Bil (mmol/L)	8.56 (5.42, 13.60)	10.00 (6.90, 18.89)	1397	0.202
D-Dimer(mg/L)	1.84 (0.98, 4.57)	3.18 (1.61, 5.43)	1252	0.066
CURB-65≥3 (points)	11/55(20.00%)	20/59(33.90%)	2.777	0.096
Respiratory failure	7/55(12.7%)	4/59(6.8%)	1.155	0.283
30-day all-cause mortality	8/55 (14.55%)	22/59(37.29%)	7.593	0.006

Underlined numbers indicates the statistical significance within this section of data.

#### 3.3.2 Survival analysis between the IG and the non-IG

**Figure 3:**
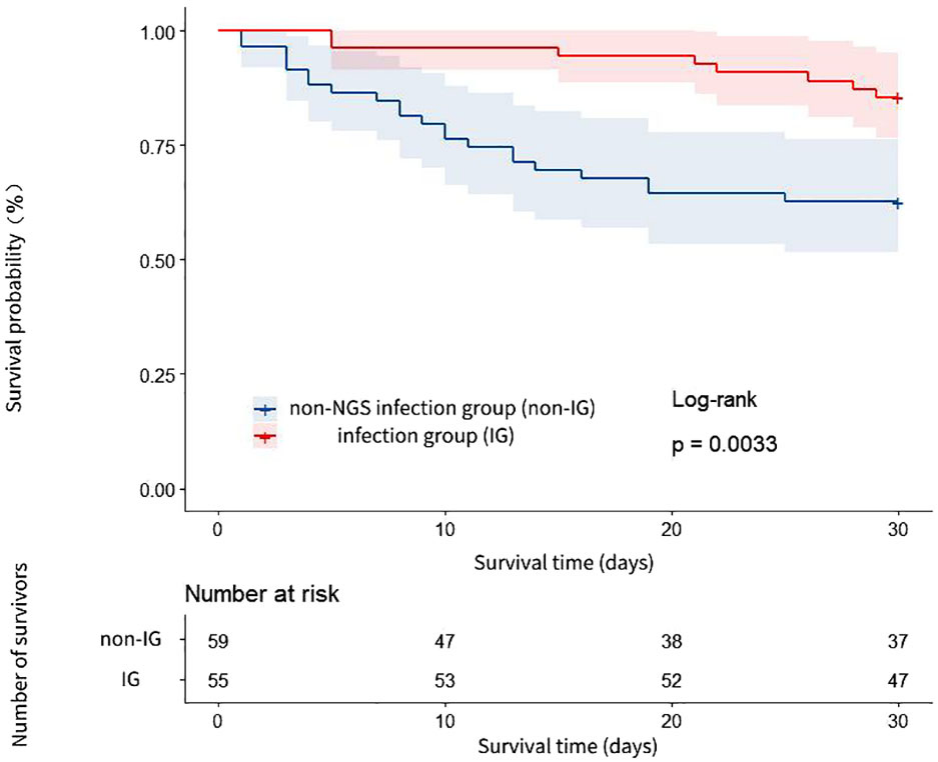
Analysis of 30-day survival curves of patients in the IG and the non-IG.

The 30-day all-cause mortality rate was significantly different between the IG and the non-IG, manifesting statistical significance (14.55% vs. 37.29%, P < 0.05, see **Table 2**). The 30-day survival rate of the IG was notably higher than that of the non-IG (HR=3.137, 95% CI: 1.531-6.427, P=0.003, see **Figure 3**).

#### 3.3.3 Analysis of prognostic factors between IG and non-IG groups

(1) Univariate Analysis: A comparative examination is conducted on the factors that might impact the prognosis of patients with diffuse pulmonary exudative lesions. These elements are subjected to COX univariate analysis, with the outcomes delineated in **Table 3**.
(2) Multifactorial Analysis: Factors exhibiting statistically significant differences (P < 0.05) from the aforementioned univariate analysis, namely: metagenomic next-generation sequencing (mNGS), underlying cardiovascular diseases, CURB-65 score ≥ 3 (which also represents age and BUN levels), and D-dimer, are incorporated as independent variables into the multifactorial Cox regression model.

**Table 3:** Univariate Cox regression analysis.

	HR	Lower. 95	Upper. 95	P-value
mNGS (yes/no)	0.318	0.141	0.714	0.006
Age (years)	1.043	1.011	1.076	0.008
Sex (male/female)	1.133	0.504	2.545	0.763
Fever (yes/no)	0.613	0.273	1.377	0.236
Cough (yes/no)	0.626	0.268	1.459	0.278
Expectoration (yes/no)	1.062	0.473	2.385	0.885
Dyspnea (yes/no)	0.449	0.183	1.099	0.079
Cardiovascular diseases (yes/no)	0.370	0.170	0.809	0.013
Chronic kidney disease (yes/no)	0.857	0.350	2.097	0.735
Chronic liver diseases (yes/no)	0.727	0.278	1.899	0.515
Tumor (yes/no)	0.616	0.236	1.609	0.322
Diabetes (yes/no)	1.250	0.536	2.914	0.605
Autoimmune rheumatic disease (yes/no)	0.673	0.275	1.647	0.386
Smoking (yes/no)	1.469	0.707	3.049	0.302
CURB-65≥3 (yes/no)	0.251	0.122	0.515	0.000
PCT (ng/ml)	1.019	0.996	1.043	0.113
CRP (mg/L)	1.000	0.996	1.004	0.945
WBC (10^9/L)	1.040	0.980	1.103	0.199
Hb (10^9/L)	0.987	0.973	1.002	0.091
BUN (mmol/L)	1.036	1.003	1.070	0.032
ALT (IU/L)	0.982	0.965	1.000	0.055
AST (IU/L)	0.997	0.990	1.004	0.380
T.Bil (mmol/L)	1.027	0.985	1.070	0.214
D-Dimer (mg/L)	1.059	1.001	1.120	0.047

Underlined numbers indicates the statistical significance within this section of data.

Initially, we coded these variables as dummy variables, followed by a collinearity diagnosis. The tolerance for each variable was substantially greater than 0.1, and the variance inflation factors were all significantly less than 10, suggesting the absence of multicollinearity among the variables (as presented in **Supplementary Table 3**). The classification of categorical variables was then delineated (as presented in **Supplementary Table 4**).

**Figure 4:**
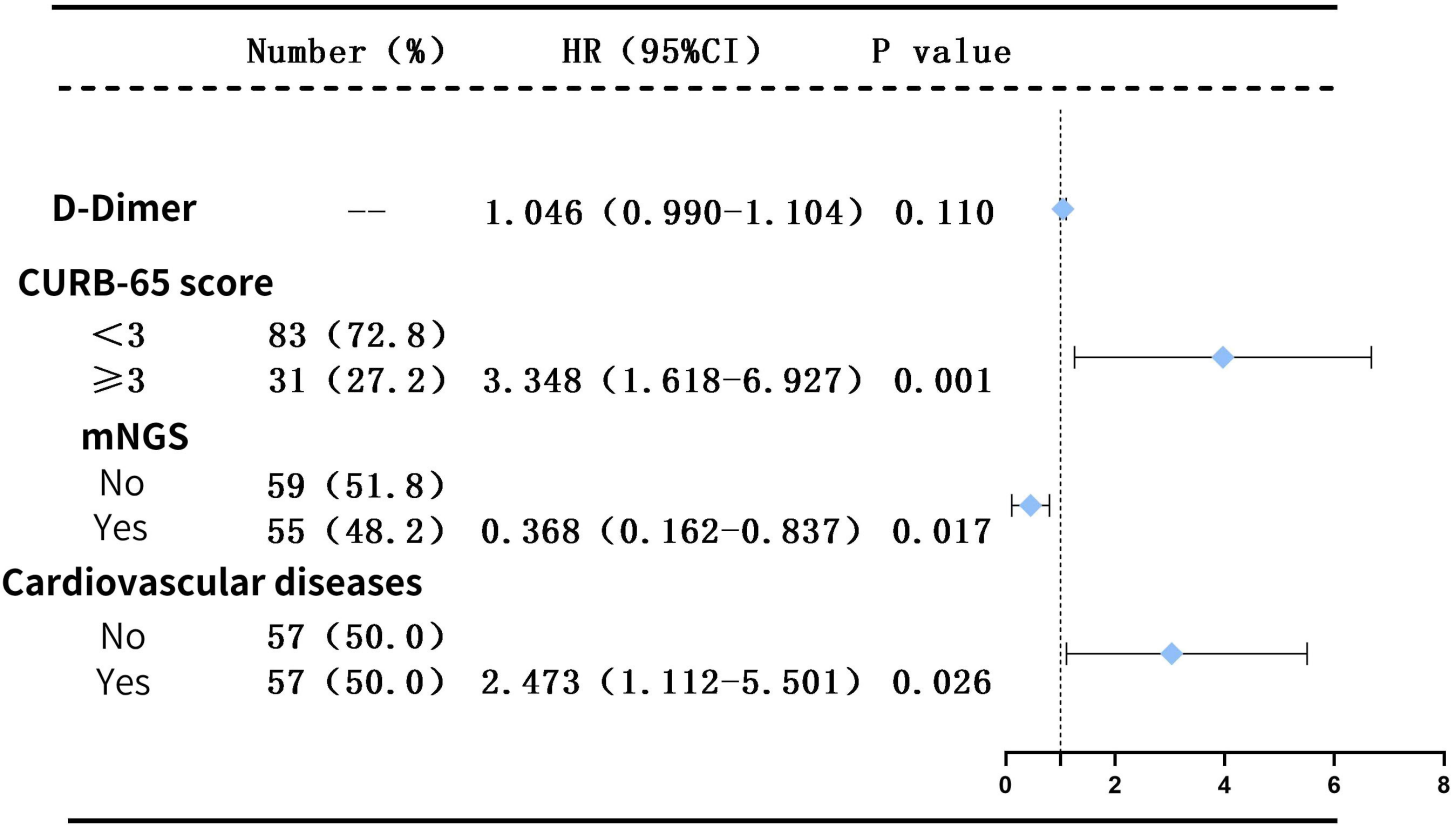
Multivariate Cox regression analysis for two Patient Cohorts.

Consequently, it was feasible to conduct a COX multivariate regression analysis. After adjusting for the confounding factors, the results still indicated that a CURB-65 score ≥3 (HR=3.348, P=0.001) and the presence of cardiovascular disease (HR=2.473, P=0.026) are independent risk factors for patients with diffuse exudative lung lesions, whereas mNGS (HR=0.368, P=0.017) serves as an independent protective factor for these patients (as illustrated in **Figure 4**).

#### 3.3.4 The efficacy analysis of mNGS in guiding the adjustment of antimicrobial therapies

In the two groups undergoing treatment, the rates of initial antimicrobial coverage for the identified pathogens were approximately equivalent (42.0% vs 47.0%, P=0.545). Within the IG and the non-IG, 32 cases (58.0%) and 31 cases (53.0%), respectively, had initial antibiotic regimens that did not encompass all detected pathogens, necessitating suitable modifications to the anti-infective strategy based on the findings. Following the adjustments to the anti-infective plans, patients’ outcomes were classified into either effective or ineffective modifications. Among the IG, the antibiotic regimen adjustments were deemed effective in 22 cases (68.75%), while the remaining 10 patients (31.25%) experienced ineffective modifications. Similarly, in the non-IG, 13 patients (41.94%) had effective regimen adjustments, as opposed to 18 patients (58.06%) with ineffective adjustments. A significant discrepancy in the rate of effective adjustments was recognized between the two cohorts (P<0.05, as detailed in **Supplementary Table 5**).

## 4 Discussion

The etiology of diffuse pulmonary exudative lesions is intricate, and the condition progresses rapidly, with numerous instances advancing to severe pneumonia. In recent years, the incidence of such patients has been on an alarming ascent, with the mortality rate remaining persistently high (Oliveira et al., 2018). Currently, the diagnostic methods employed in the clinical arena for the assessment of diffuse pulmonary exudative lesions are of limited efficacy, necessitating more comprehensive tools with superior diagnostic capabilities to enhance the management and treatment standards for these patients.

Our investigation contrasts the diagnostic efficacy of mNGS with traditional culture methods by examining their pathogen detection results. And the aim is to discern, expeditiously, during the disease’s incipient stages whether pulmonary diffuse exudative lesions are indicative of an infectious etiology, and to rapidly identify the causative pathogens, thereby facilitating early, targeted treatment. Additionally, we endeavor to appraise the impact of mNGS technology on the prognosis of patients with infectious pulmonary diffuse exudative lesions to ascertain the utility of this technology in the management of such diseases. Additionally, we endeavor to appraise the impact of mNGS technology on the prognosis of patients with infectious pulmonary diffuse exudative lesions to ascertain the utility of this technology in the management of such diseases.

Despite the availability of numerous and mature detection methodologies, approximately half of patients with severe infections still cannot be definitively diagnosed etiologically (Schlaberg et al., 2017; Wu et al., 2023). Our research findings indicate that the sensitivity of pathogen detection using mNGS stands at an impressive 80.0%. In comparison to traditional culture methods, mNGS demonstrates enhanced sensitivity in diagnosing infectious pulmonary diffuse exudative lesions by 34.5%, corroborating previous studies (Miao et al., 2018). Upon incorporating results from other pathogen detection methods, such as serological experiments and PCR, we found that these results supported the mNGS detection results in the IG group by 100%. In contrast, the support rates for culture-based methods in the IG group and non-IG group were only 0% (P=0.029) and 33% (P=0.026) respectively. This also suggests a greater advantage of mNGS in diagnosing infectious diffuse pulmonary infiltrates. Moreover, the utility of mNGS is even more pronounced in identifying mixed and specific infections (refer to **Supplementary Table 6**, showing significant differences for mixed infections and specific infections, with P-values < 0.001 and P = 0.004, respectively), aligning with findings reported in extant literature (Wang et al., 2019a; Sun et al., 2021). Further scrutiny of patient data concerning these mixed and specific infections revealed that the majority conformed to criteria for severe pneumonia. A multi-center study (Wu et al., 2020) highlighted that the detection rate of mixed infections in patients with severe community-acquired pneumonia (SCAP) reached 56.8%, where mNGS demonstrated a markedly higher positivity rate than other laboratory methodologies (culture, serological methods, and PCR). This underscores the likelihood that patients presenting with diffuse exudative pulmonary lesions, especially those exhibiting severity consistent with severe pneumonia, may be suffering from mixed infections. For such patients, mNGS emerges as a more excellent and comprehensive diagnostic option.

It is noteworthy that in recent years, with the clinical application of mNGS, an increasing number of cases of pneumonia of unknown etiology have been attributed to infection with Chlamydia psittaci (X et al., 2022). Owing to its unique biphasic life cycle, Chlamydia psittaci frequently leads to multiorgan dysfunction and severe infections. Radiologically, it may present with diffuse exudative changes in both lungs. The onset is abrupt, and the progression rapid. If diagnosis and treatment are not timely, it can result in fatality (Kong et al., 2021). In this particular study, mNGS identified five patients with Chlamydia psittaci (see **Supplementary Table 7**), all of whom, following the adjustment of their targeted anti-infection treatment based on the findings, exhibited significant improvement and were subsequently discharged in better health. This suggests that mNGS should be considered for prompt pathogen detection in future cases of pneumonia with unknown causes (Geng et al., 2021; Qian et al., 2021).

Moreover, our study also explored the impact of antibiotic exposure on the detection rates via two distinct methodologies. The results indicated that the mNGS outcomes were less affected by antibiotic exposure. A possible explanation for this could be that antibiotic exposure suppresses or eradicates a portion of the pathogenic microorganisms present in the sample, leading to a significantly reduced positivity rate in culture-based methods. However, since mNGS detects the nucleic acid sequences of all pathogens, it remains unaffected by the state of the pathogens (Diao et al., 2022).

In patients suffering from infectious pulmonary diffuse exudative lesions, the condition progresses rapidly and a significant proportion are afflicted with severe pneumonia. Despite aggressive and comprehensive antibacterial treatments (Martin-Loeches et al., 2023), a high mortality rate persists. Thus, we investigated the prognostic value of mNGS in these patients. Baseline data before initial treatment in both the IG and the non-IG were comparable, as was the severity of their conditions. However, after treatment, the two groups exhibited a significant difference in 30-day all-cause mortality (15.55% vs 37.29%, P=0.006), with the risk of death nearly halved. After conducting COX univariate and multivariate regression analyses and adjusting for confounding factors, mNGS testing (HR=0.368, P=0.017) was indicated as an independent protective factor for patients with pulmonary diffuse exudative lesions. To further explore the reasons, we analyzed the effectiveness of antimicrobial treatment adjustments in both IG and non-IG. Based on the comparably proportional coverage of initial antimicrobial treatment of the pathogens between the two groups (approximately 42.0% versus 47.0%, P=0.545), subsequent adjustments to the anti-infective regimen were made in accordance with the examinations, with the effectiveness rate being assessed by the ultimate clinical outcome of the diseases. In the IG, the rate of effective antibiotic regimen adjustment was 68.75%, significantly higher than the 41.94% seen in the non-IG (P<0.05). This suggests that mNGS testing allows for better targeted antimicrobial treatment and may be a key factor in improving clinical outcomes.

Peng Zhang (Diao et al., 2022) et al. conducted an analysis on 95 patients suffering from acute respiratory distress syndrome (ARDS) caused by severe pneumonia. By assessing APACHE II and SOFA scores over a period of seven consecutive days, they dynamically evaluated the therapeutic efficacy of antimicrobial treatments guided by mNGS and traditional detection methods, thus substantiating that mNGS serves as an independent protective factor for patients with severe pneumonia-induced ARDS. Additionally, Yun Xie (Xie et al., 2019) et al. analyzed 178 cases of severe pneumonia, uncovering that when treatment was guided by the results of mNGS testing, there was a significant improvement in the 28-day and 90-day survival rates of patients with severe pneumonia. These studies collectively suggest that mNGS testing can lead to a more favorable prognosis for severe pneumonia.

The present study focuses on a distinct cohort of patients manifesting with diffuse pulmonary exudative lesions, encompassing clinical scenarios such as interstitial lung abnormalities due to connective tissue disease, as well as severe infectious pulmonary exudative conditions. These individuals, at the time of their initial consultation, frequently present with a clinical conundrum that obscures the differentiation between infectious and non-infectious etiologies of their ailments. The research uncovered that not only does mNGS offer considerable diagnostic utility, but it also revealed that early detection aids in determining whether pulmonary diffuse exudative lesions are infectious. Furthermore, early application of mNGS can rapidly supply a definitive pathogen spectrum for infections causing diffuse exudative lesions, thereby facilitating precise and early targeted anti-pathogen treatments, which could be a critical factor in enhancing patient prognoses.

In conclusion, the mNGS technology exhibits a higher sensitivity in the etiological diagnosis of patients with diffuse pulmonary exudative lesions, with reduced interference from antibiotics. It is a vital instrument for the clinical diagnosis of the etiology behind diffuse pulmonary exudative lesions. Early application of mNGS can assist in the prompt etiological diagnosis of such diseases and play a crucial role in guiding clinical treatment decisions. However, this study has several limitations: First, it is a single-center study with a limited sample size, raising the possibility of selection bias and limiting the generalizability of the results. And the performance of traditional methods may be underestimated due to the lack of complete results from other conventional diagnostic techniques, such as serological testing and PCR. Second, as this study is retrospective, most of the patient data in non-IG primarily relies on sputum culture results as the gold standard. Other results, such as those from serological experiments and PCR, lack consistency among patients and have significant omissions, making it currently impossible to supplement them or systematically analyze all the test results together. And the retrospective nature of the study led to the exclusion of cases lost to follow-up. And it also solely investigates final outcomes, lacking criteria for assessing the dynamic progression of the illness and variations in the intensity of antimicrobial medication usage. Future research should thus involve larger-scale prospective multicenter trials to further investigate these matters. Third, the specimens analyzed in this study were primarily bronchoalveolar lavage fluids, and the scarcity of other types of specimens precludes an assessment of detection rate discrepancies among different samples. Hence, future studies should include a greater variety of specimens to explore potential differences in detection rates.

## Data availability statement

The original contributions presented in the study are included in the article/**Supplementary Material**. Further inquiries can be directed to the corresponding authors.

## Ethics statement

The studies involving human participants were reviewed and approved by the Ethics Committee of Fujian Provincial Hospital (ethics number: K2019-12-032). The studies were conducted in accordance with the local legislation and institutional requirements. The participants provided their written informed consent to participate in this study.

## Author contributions

YW: Writing – original draft. JW: Writing – original draft. NX: Writing – review & editing. ML: Writing – review & editing. WY: Writing – review & editing. YC: Writing – review & editing. QZ: Writing – review & editing. HL: Writing – review & editing.
