# Supplementary material for: Clinical application value of metagenome next-generation sequencing in pulmonary diffuse exudative lesions: a retrospective study
Source: Front Cell Infect Microbiol. 2024 May 8;14:1367885. doi: 10.3389/fcimb.2024.1367885 (PMC11113015; doi:10.3389/fcimb.2024.1367885)
Supplement: Supplementary file 1 [file DataSheet_1.docx]

Table 1 Comparative Analysis of Baseline Characteristics Between the IG and non-IG

|  | **IG**  **（n=55）** | **Non-IG**  **（n=59）** | **χ^2^/t/U value** | **P-value** |
| --- | --- | --- | --- | --- |
| Age | 65.00  （57.00，72.00） | 67.00  （56.00，75.00） | 1486 | 0.439 |
| Sex |  |  |  |  |
| Male | 41/55（74.55%） | 44/59（74.58%） | 1.425 | 0.997 |
| Female | 14/55（25.45%） | 15/59（25.42%） |  |  |
| Cardiovascular diseases | 24/55（43.64%） | 33/59（55.93%） | 1.721 | 0.190 |
| Chronic kidney disease | 10/55（18.18%） | 10/59（16.95%） | 0.030 | 0.863 |
| Chronic liver diseases | 10/55（18.18%） | 5/59（8.47%） | 2.347 | 0.126 |
| Tumor | 8/55（14.55%） | 6/59（10.17%） | 0.506 | 0.477 |
| Diabetes | 16/55（29.09%） | 14/59（23.73%） | 0.422 | 0.516 |
| Autoimmune rheumatic  disease | 9/55（16.36%） | 9/59（15.25%） | 0.026 | 0.871 |
| Smoking | 25/55（45.45%） | 29/59（49.15%） | 0.156 | 0.693 |

Table 2 The distribution of disease diagnoses within the NIG group.

|  | Final diagnosis（NIG） |
| --- | --- |
| **ILDs of identified cause** |  |
| CTDs | 11 |
| HP | 1 |
| Latrogenic ILDs（drug-induced） | 1 |
| **IIPs** |  |
| IPF | 1 |
| Acute interstitial pneumonia | 1 |
| Unclassifiable IIPs | 6 |
| **Sarcoid-like granulomatous diseases** |  |
| **Other ILDs** |  |
| Pulmonary amyloidosis | 1 |

Notes: CTDs: connective tissue diseases; HP: Hypersensitivity pneumonitis; IIPs: idiopathic interstitial pneumonias; IPF: idiopathic pulmonary fibrosis

Table 3 Collinearity diagnosis of risk factors.

| **Variable** | **Tolerance** | **VIF** |
| --- | --- | --- |
| mNGS | 0.961 | 1.040 |
| CURB-65≥3 | 0.963 | 1.039 |
| Cardiovascular diseases | 0.978 | 1.022 |
| D-Dimer | 0.989 | 1.011 |

Table 4. Assignment table.

| **Variable** | **Assignment** |
| --- | --- |
| mNGS | Yes=1，No=0 |
| CURB-65≥3 | Yes=1，No=0 |
| Cardiovascular diseases | Yes=1，No=0 |

Table 5. Comparison of adjustment efficiency.

|  | **Effective** | **Ineffective** | **χ^2^值** | **P值** |
| --- | --- | --- | --- | --- |
| IG | 22 | 10 | 4.585 | 0.032 |
| non-IG | 13 | 18 |  |  |

Table 6. Distribution of detection results of mNGS and culture.

|  | **IG** | |  | **non-IG** |
| --- | --- | --- | --- | --- |
|  | mNGS | culture |  | culture |
| **Bacteria** |  |  |  |  |
| Streptococcus pneumoniae | 5 | 1 |  | 0 |
| Haemophilus influenzae | 1 | 0 |  | 0 |
| Klebsiella pneumoniae | 2 | 6 |  | 4 |
| Pseudomonas aeruginosa | 5 | 3 |  | 2 |
| Staphylococcus aureus | 1 | 0 |  | 4 |
| Escherichia coli | 1 | 1 |  | 1 |
| Acinetobacter baumannii | 1 | 5 |  | 10 |
| Enterococcus | 4 | 1 |  | 0 |
| Other Streptococcal bacteria | 5 | 4 |  | 0 |
| stenotrophomonas maltophilia | 3 | 0 |  | 2 |
| Corynebacterium striatum | 2 | - |  | - |
| Other rare bacteria | 15 | 2 |  | 1 |
| **fungus** |  |  |  |  |
| Candida albicans | 7 | 5 |  | 19 |
| Nocardia otitidiscaviarum | 1 | - |  | - |
| aspergillus | 4 | 0 |  | 1 |
| Pneumocystis jirovecii | 8 | 1 |  | 0 |
| **virus** |  |  |  |  |
| EB virus | 4 | - |  | - |
| cytomegalovirus | 3 | - |  | - |
| **Specific pathogens** |  |  |  |  |
| mycobacterium tuberculosis | 3 | 1 |  | 0 |
| nontuberculous mycobacteria | 5 | 2 |  | 0 |
| chlamydia psittaci | 5 | - |  | 0 |
| legionella pneumophila | 2 | - |  | 0 |
| **total** | 87 | 32 |  | 44 |

Table 7. Comparison of positivity rate of infection types between mNGS and culture.

|  | **mNGS** | **Culture** | **χ^2^** | **P-value** |
| --- | --- | --- | --- | --- |
| Only bacteria | 16（20.78%） | 19（24.68%） | 0.333 | 0.564 |
| Only virus | 0（0%） | 0（0%） | / | 1.000* |
| Only fungi | 4（5.19%） | 4（5.19%） | 0.132 | 0.717 |
| Mixed infections | 15（19.48%） | 1（1.30%） | 11.79 | ＜0.001 |
| Specific infections | 14（18.18%） | 2（2.60%） | 8.439 | 0.004 |

Note: The symbol *P represents the exact probability as determined by Fisher’s method.

Table 8 A comparison of the consistency in pathogen detection results between the IG and the non-IG using traditional culturing methods

|  | **IG** | **Non-IG** | **χ^2^值** | **P^*^值** |
| --- | --- | --- | --- | --- |
| Bacteria only | 16（29.1%） | 19（22.0%） | 8.213 | 0.062 |
| Virus only | 0（0%） | 0（0%） |  |  |
| Fungi only | 5（9.1%） | 14（23.7%） |  |  |
| mixed infection | 2（3.6%） | 6（4.1%） |  |  |
| Specific infection | 2（3.6%） | 0（0%） |  |  |
| Not detected | 30（54.5%） | 26（44.1%） |  |  |

Note: *P is fisher's exact test. Specific infection include Mycobacterium tuberculosis, non-tuberculous mycobacteria, Legionella pneumophila, and Chlamydophila psittaci.

Table 9 The support rate for pathogen detection results between the IG group and non-IG group using alternative detection methods

|  | **IG** | | **P** | **non-IG** | **P** |
| --- | --- | --- | --- | --- | --- |
|  | mNGS | culture |  | culture |  |
| other detection methods (such as serological experiments and PCR) | 4 | |  | 21 |  |
| The results support pathogen diagnosis. | 4（100%） | 0（0%） | 0.029 | 7（33%） | 0.026 |


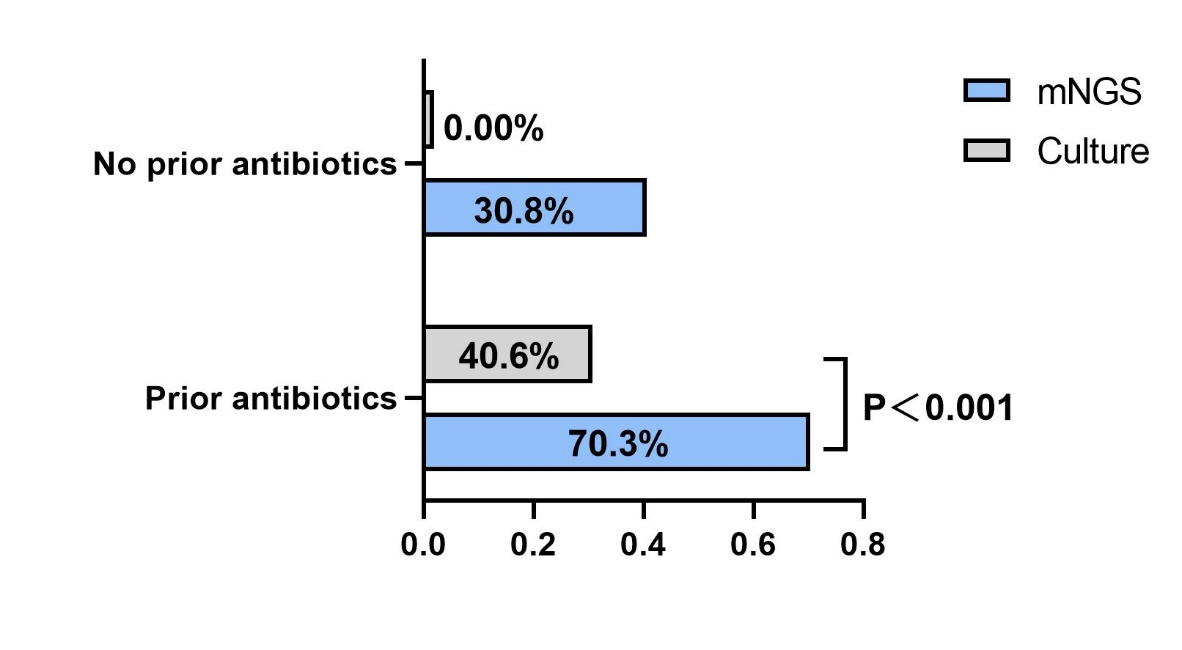
Fig.1.The effect of antibiotic exposure on mNGS positivity.

Clarification: mNGS reveals its clinical importance in real-world settings. The proportions displayed in the figure above represent the positivity rates detected by the respective methods for each group. Within samples exposed to antibiotics, the positivity rate of mNGS is notably superior to that of traditional culture (P < .01), indicating that mNGS is less susceptible to the effects of antibiotic exposure.
